# Supplementary material for: Outcomes and outcome measures reported in clinical studies of therapeutic mammaplasty: a systematic review protocol
Source: BMJ Open. 2021 Jun 16;11(6):e046438. doi: 10.1136/bmjopen-2020-046438 (PMC8211071; doi:10.1136/bmjopen-2020-046438)
Supplement: Supplementary data [file bmjopen-2020-046438supp002.pdf]

Supplementary material: Search strategies

Ovid EMBASE

1. exp breast cancer/
2. breast neoplasm*.mp.
3. (breast adj2 cancer*).mp.
4. (breast adj2 tumo?r*).mp.
5. 1 or 2 or 3 or 4
6. (therapeutic adj3 mamm?plast*).mp.
7. reduction mamm?plast*.mp.
8. oncoplastic breast surger*.mp.
9. breast reconstruction/
10. 6 or 7 or 8 or 9
11. 5 and 10
12. clinical trial.de.
13. randomization.de.
14. crossover procedure.de.
15. randomized controlled trial.de.
16. single blind procedure.de.
17. double blind procedure.de.
18. placebo.de.
19. prospective study.de.
20. (randomi?ed controlled adj1 trial*).mp. [mp=title, abstract, heading word, drug trade name, original title, device manufacturer, drug manufacturer, device trade name, keyword, floating subheading word, candidate term word]
21. rct.mp. [mp=title, abstract, heading word, drug trade name, original title, device manufacturer, drug manufacturer, device trade name, keyword, floating subheading word, candidate term word]
22. (random* adj1 allocat*).mp. [mp=title, abstract, heading word, drug trade name, original title, device manufacturer, drug manufacturer, device trade name, keyword, floating subheading word, candidate term word]
23. (single adj1 blind*).mp. [mp=title, abstract, heading word, drug trade name, original title, device manufacturer, drug manufacturer, device trade name, keyword, floating subheading word, candidate term word]

24. (double adj1 blind*).mp. [mp=title, abstract, heading word, drug trade name, original title, device manufacturer, drug manufacturer, device trade name, keyword, floating subheading word, candidate term word]
25. ((treble or triple) adj1 (blind* or placebo*)).mp. [mp=title, abstract, heading word, drug trade name, original title, device manufacturer, drug manufacturer, device trade name, keyword, floating subheading word, candidate term word]
26. 12 or 13 or 14 or 15 or 16 or 17 or 18 or 19 or 20 or 21 or 22 or 23 or 24 or 25
27. exp cohort analysis/
28. exp longitudinal study/
29. exp prospective study/
30. exp follow up/
31. cohort$.tw.
32. exp case control study/
33. (case$ and control$).tw.
34. 27 or 28 or 29 or 30 or 31 or 32 or 33
35. 26 or 34
36. 11 and 35

Ovid Medline

1. (therapeutic adj3 mamm?plast*).mp.
2. reduction mamm?plast*.mp.
3. oncoplastic breast surger*.mp.
4. Mammaplasty/
5. 1 or 2 or 3 or 4
6. exp Breast Neoplasms/
7. breast neoplasm*.mp.
8. (breast adj2 cancer*).mp.
9. (breast adj2 tumo?r*).mp.
10. 6 or 7 or 8 or 9
11. 5 and 10
12. randomized controlled trial.pt.
13. controlled clinical trial.pt.
14. randomi?ed.ab.

15. placebo.ab.
16. drug therapy.fs.
17. randomly.ab.
18. trial.ab.
19. groups.ab.
20. 12 or 13 or 14 or 15 or 16 or 17 or 18 or 19
21. exp cohort studies/
22. cohort$.tw.
23. controlled clinical trial.pt.
24. epidemiologic methods/
25. limit 24 to yr="1966 - 1989"
26. exp case-control studies/
27. (case$ and control$).tw.
28. 21 or 22 or 23
29. 24 or 25 or 26 or 27
30. 28 or 29
31. 20 or 30
32. 11 and 31

CINAHL

1. MH Breast Neoplasms+
2. Breast neoplasm*.mp
3. (breast adj2 cancer*).mp.
4. 1 or 2 or 3
5. therapeutic adj3 mamm?plast*.mp
6. oncoplastic breast surger*
7. reduction mamm?plast*
8. breast reconstruction/
9. 5 or 6 or 7 or 8
10. 4 and 9

Web of Science

ALL=((breast neoplasm* OR (breast "NEAR" cancer*) OR (breast "NEAR" tumo?r*)) AND (therapeutic "NEAR" mamm?plast* OR reduction mamm?plast* OR oncoplastic breast surger*))
