# Supplementary material for: Outcomes and outcome measures reported in clinical studies of therapeutic mammaplasty: a systematic review protocol
Source: BMJ Open. 2021 Jun 16;11(6):e046438. doi: 10.1136/bmjopen-2020-046438 (PMC8211071; doi:10.1136/bmjopen-2020-046438)
Supplement: Author's manuscript [file bmjopen-2020-046438.draft_revisions.pdf]

BMJ Open

BMJ Open is committed to open peer review. As part of this commitment we make the peer review history of every article we publish publicly available.

When an article is published we post the peer reviewers' comments and the authors' responses online. We also post the versions of the paper that were used during peer review. These are the versions that the peer review comments apply to.

The versions of the paper that follow are the versions that were submitted during the peer review process. They are not the versions of record or the final published versions. They should not be cited or distributed as the published version of this manuscript.

BMJ Open is an open access journal and the full, final, typeset and author-corrected version of record of the manuscript is available on our site with no access controls, subscription charges or pay-per-view fees (http://bmjopen.bmj.com).

If you have any questions on BMJ Open's open peer review process please email info.bmjopen@bmj.com

BMJ Open

Outcomes and outcome measures reported in clinical studies of therapeutic mammaplasty: A systematic review protocol

Journal:	*BMJ Open*
Manuscript ID	bmjopen-2020-046438
Article Type:	Protocol
Date Submitted by the Author:	03-Nov-2020
Complete List of Authors:	Lee, Alice; Imperial College London, Department of Surgery and Cancer<br>Kwasnicki, Richard; Imperial College London, Department of Surgery and Cancer<br>Leff, Daniel; Imperial College London, Department of Surgery and Cancer
Keywords:	Plastic & reconstructive surgery < SURGERY, Breast surgery < SURGERY, STATISTICS & RESEARCH METHODS

SCHOLARONE™
Manuscripts

I, the Submitting Author has the right to grant and does grant on behalf of all authors of the Work (as defined in the below author licence), an exclusive licence and/or a non-exclusive licence for contributions from authors who are: i) UK Crown employees; ii) where BMJ has agreed a CC-BY licence shall apply, and/or iii) in accordance with the terms applicable for US Federal Government officers or employees acting as part of their official duties; on a worldwide, perpetual, irrevocable, royalty-free basis to BMJ Publishing Group Ltd ("BMJ") its licensees and where the relevant Journal is co-owned by BMJ to the co-owners of the Journal, to publish the Work in this journal and any other BMJ products and to exploit all rights, as set out in our licence.

The Submitting Author accepts and understands that any supply made under these terms is made by BMJ to the Submitting Author unless you are acting as an employee on behalf of your employer or a postgraduate student of an affiliated institution which is paying any applicable article publishing charge ("APC") for Open Access articles. Where the Submitting Author wishes to make the Work available on an Open Access basis (and intends to pay the relevant APC), the terms of reuse of such Open Access shall be governed by a Creative Commons licence – details of these licences and which Creative Commons licence will apply to this Work are set out in our licence referred to above.

Other than as permitted in any relevant BMJ Author's Self Archiving Policies, I confirm this Work has not been accepted for publication elsewhere, is not being considered for publication elsewhere and does not duplicate material already published. I confirm all authors consent to publication of this Work and authorise the granting of this licence.

For peer review only

Outcomes and outcome measures reported in clinical studies of therapeutic mammaplasty: A systematic review protocol

For peer review only

Alice Lee[1], Richard M Kwasnicki[1], Daniel Leff[1]

Author affiliations:

1. Department of Surgery and Cancer, Faculty of Medicine, Imperial College London, South Kensington Campus, London SW7 2AZ, UK

Corresponding author:

Dr Alice Lee

Address: Department of Surgery and Cancer, Faculty of Medicine, Imperial College London, South Kensington Campus, London SW7 2AZ, UK

Email: alice.lee13@imperial.ac.uk

For peer review only - http://bmjopen.bmj.com/site/about/guidelines.xhtml

Abstract

Introduction

Therapeutic mammaplasty (TM) is an oncological procedure which combines tumour resection with breast reduction and mastopexy techniques. Previous systematic reviews have demonstrated oncological safety of TM, but poor and inconsistent reporting of quality of life, aesthetic and functional outcomes, often with non-validated measurement tools. Moreover, there is a paucity of patient-reported outcome measures. Standardisation of outcome reporting is required to enable study results to be compared and combined, for example through Core Outcome Set (COS) development. This systematic review aims to comprehensively describe the outcomes reported in clinical studies of TM, their respective outcome measures and the time points at which they were evaluated. The overall objective is to facilitate the development of a COS for TM.

Methods and analysis

A systematic review of clinical studies evaluating outcomes following TM will be completed according to the Preferred Reporting Items for Systematic Reviews and Meta-Analyses (PRISMA) guidelines. The following electronic databases have been searched from inception to 05/08/2020: Ovid Medline, EMBASE, CINAHL and Web of Science. Primary outcomes will include the number of reported outcomes of various types (clinical, aesthetic, functional, quality-of-life and cost-effectiveness), whether these are patient- or clinician-reported, how outcomes are defined and the outcome measurement tool(s) used. The time point(s) at which outcomes were measured will be a secondary outcome. No studies will be excluded on the basis of methodological quality in order to generate a comprehensive list of reported outcomes and outcome measures; hence, risk of bias assessment is not required. The data will be described narratively. This protocol has been reported in line with PRISMA-P.

For peer review only

Ethics and dissemination

This study does not involve human or animal participants, hence ethical approval is not required. The findings will be published in a peer-reviewed journal and presented at relevant conferences.

Registration: This review is registered in PROSPERO (CRD42020200365).

Word count: 300

Keywords: therapeutic mammaplasty; core outcome set; outcome measurement; patient-reported outcomes

For peer review only

Article Summary

Strengths and limitations of this study (up to 5 bullet points)

- This systematic review will facilitate the development of the first Core Outcome Set for therapeutic mammaplasty.
- A Core Outcome Set will improve the synthesis and meta-analysis of new and existing research into therapeutic mammaplasty.
- The systematic review described in this protocol will build on existing literature by providing an updated review of outcomes following therapeutic mammaplasty with a broader scope, including clinical, aesthetic, functional, quality-of-life and patient-reported outcomes.
- The literature search was limited to English language articles, which may have excluded some relevant papers.

For peer review only

For peer review only - http://bmjopen.bmj.com/site/about/guidelines.xhtml

Introduction

Background

Therapeutic mammaplasty (TM) is an oncological procedure which combines cancer resection with breast reduction and mastopexy techniques [1]. TM can facilitate breast-conserving surgery (BCS) for larger tumours [2] to safely avoid mastectomy [3] and improve cosmesis in cases where standard BCS would otherwise have poor outcomes [4]. TM may also minimise radiotherapy-related side effects in women with larger breasts [4,5] and be a favourable option for women with pre-existing macromastia who seek the functional and psychological benefits of breast reduction surgery [4]. Previous systematic reviews have suggested satisfactory oncological safety of TM [2,4,6], but poor and inconsistent reporting of quality of life, aesthetic and functional outcomes, often with non-validated measurement tools [5,7,8]. Moreover, there is a paucity of patient-reported outcome measures [6].

A Core Outcome Set (COS) describes the minimum number of outcomes to be reported across all trials of one healthcare domain [9]. This reduces the heterogeneity of outcome reporting across trials, allowing results to be compared and combined in meta-analyses, to inform best medical practice [9]. TM is becoming routine practice in oncoplastic breast units, however there is no standardised way to evaluate outcomes following this procedure which incorporates the views of both healthcare professionals and patients as stakeholders. A related COS on reconstructive breast surgery [10] mainly focussed on post-mastectomy reconstruction; only 10% of patient stakeholders in the project had undergone TM and some outcomes included in the final COS (e.g., implant related complications) are less relevant to the TM population. There is good reason to hypothesise that TM patients may evaluate and prioritise their treatment outcomes differently to patients undergoing other forms of breast reconstruction. For example, improved functional outcomes associated with breast reduction techniques and avoidance of mastectomy may significantly drive treatment decisions.

For peer review only - http://bmjopen.bmj.com/site/about/guidelines.xhtml

Rationale

In order to develop a COS for TM, a comprehensive systematic review of all available outcomes and outcome measures reported in the literature is required. The systematic review described in this protocol is the first stage in the development of a COS for TM, which is planned and has been prospectively registered on the Core Outcome Measures in Effectiveness Trials (COMET) database (http://comet-initiative.org/Studies/Details/1655).

Aims and objectives

Review aims

The overall aim of this systematic review is to identify all outcomes and outcome measures used to evaluate TM in the literature and how the authors define these outcomes. The timepoints at which these outcomes are measured will be a secondary outcome.

Objectives

The specific objectives of this review are to analyse all clinical studies of TM in adult, female participants in order to:

i) Identify the number of unique outcomes and outcome measures reported;
ii) Identify and describe variation in outcome definitions;
iii) Identify and describe variation in the timepoint(s) used to measure outcomes;
iv) Identify the number of different types of outcome (clinical, aesthetic, functional, quality-of-life and cost-effectiveness outcomes) reported per study and across all included studies and whether these are clinician- or patient-reported;
v) Group unique outcomes into domains, to facilitate the development of a COS for TM.

For peer review only

Methods and analysis

This protocol has been developed in accordance with the PRISMA-P guidelines [11]. A PRISMA-P checklist for this protocol can be found in the **supplementary material**. The systematic review described in this protocol has been prospectively registered on PROSPERO (CRD42020200365). Any amendments to this protocol, involving screening, data extraction or data synthesis will be documented and referenced in any subsequent, related publications.

Eligibility criteria

This systematic review will include clinical studies of adult, female participants who have undergone TM as primary treatment for breast cancer. For the purpose of this systematic review, TM will be defined as the use of oncoplastic reduction or mastopexy techniques, including removal of the skin envelope and/or nipple if indicated, to treat pre-invasive or invasive breast cancer with BCS [12]. The eligibility criteria are summarised in **Table 1**. At the time of writing this protocol, screening (by title and abstract) has commenced. The initial search returned 5709 de-duplicated articles.

Information sources

The following electronic databases have been searched from inception to 05/08/2020: Ovid Medline, EMBASE, CINAHL and Web of Science. The reference lists of included articles will also be hand-searched. The outcomes generated from this review will be cross-referenced with those reported in the National Mastectomy and Breast Reconstruction Audit [13] and Oncoplastic Breast Reconstruction Guidelines for Best Practice co-produced by the Association of Breast Surgery and British Association of Plastic Reconstructive and Aesthetic Surgeons [14]. Outcomes from these national documents which are relevant to the TM population and not already included in the review will be added.

Search strategy

For peer review only - http://bmjopen.bmj.com/site/about/guidelines.xhtml

An example search strategy for Ovid Medline is provided in **Table 2**. The search strategies for EMBASE, CINAHL and Web of Science can be found in the **supplementary material**. In order to focus the search and make screening numbers manageable, validated study design filters for clinical trials, cohort studies and case-control studies were used [15,16].

Study records

Covidence software (v2103) will be used for de-duplication of citations and article screening. Two researchers will independently screen papers against inclusion criteria in two stages: i) by title and abstract, and ii) by full text. Data will be extracted using a piloted form in Microsoft Excel (v16.41), developed for the purposes of this review. Included studies will be extracted independently by two researchers. The first 10 included full texts will be extracted to trial the suitability of the data extraction sheet. Any conflicts which arise in the screening or extraction stages will be resolved through discussion between the relevant researchers; if they cannot be resolved a third researcher (RMK or DL) will make the final decision.

Data items

The following variables will be extracted: study details (publication year, study design, TM procedure, average follow-up time) and information on study population (n number and average age). Outcome data will include total number of reported outcomes, whether outcomes are clinician- or patient-reported, how study authors have defined each outcome, the outcome measure(s) used and the time point(s) at which each outcome is measured. Outcomes will be categorised (into clinical, aesthetic, functional, quality-of-life and cost-effectiveness outcomes) by the researchers performing data extraction. Clinical outcomes will include oncological outcomes and operative complications. Aesthetic outcomes will include all measures of satisfaction with post-operative appearance, either clinician- or patient-reported. Functional outcomes will include, but are not limited to, level of physical activity, neck, shoulder, breast or back pain and intertrigo. Quality-of-life outcomes will include

For peer review only

domains such as psychosocial, sexual and physical wellbeing. Cost-effectiveness outcomes will include formal analysis of cost-effectiveness or surrogate measures such as length of stay. Outcome measures which fall into more than one of the aforementioned categories will be reported as such and described narratively.

Outcomes and prioritisation

The primary outcomes of this study will include the number of unique outcomes and outcome measures reported in studies of TM and any reported variation in outcome definition between studies. As a secondary outcome, we will summarise the timepoints used for outcome measurement and any variation between studies with respect to the timing of outcome measurement.

Risk of bias in individual studies

No studies will be excluded on the basis of methodological quality in order to generate a comprehensive list of reported outcomes and outcome measures; hence, risk of bias assessment is not required.

Data synthesis

The following data will be summarised quantitively: overall number of outcomes per study; number of outcomes categorised as clinical, aesthetic, quality-of-life and cost-effectiveness per study; the number of different definitions used for each outcome across all included studies and the number of different outcome measures and time points used to measure each outcome across all included studies. The percentage of included studies which evaluate aesthetic, functional, quality-of-life, cost-effectiveness and patient-reported outcomes will be calculated. Outcomes will then be grouped into domains using an existing [9] or author-generated ontological framework, depending on the final list of outcomes obtained.

For peer review only

Ethics and dissemination

This study does not involve human or animal participants, hence ethical approval is not required.

The completed systematic review will be presented at relevant academic conferences, reported in accordance with PRISMA guidelines [17] and published in a peer-reviewed journal.

Patient and public involvement

Patients were not directly involved in the design or conduct of this systematic review, since no participant recruitment will take place and the research is based on previously published data.

For peer review only

For peer review only - http://bmjopen.bmj.com/site/about/guidelines.xhtml

References

1 Currie A, Chong K, Davies GL. Using therapeutic mammoplasty to extend the role of breast-conserving surgery in women with larger or ptotic breasts. *Ann R Coll Surg Engl* Published Online First: 2013. doi:10.1308/003588413X13511609958091

2 Schaverien M V., Raine C, Majdak-Paredes E, *et al.* Therapeutic mammaplasty-Extending indications and achieving low incomplete excision rates. *Eur J Surg Oncol* Published Online First: 2013. doi:10.1016/j.ejso.2013.01.006

3 O'Connell RL, Rattay T, Dave R V, *et al.* The impact of immediate breast reconstruction on the time to delivery of adjuvant therapy: the iBRA-2 study. *Br J Cancer* 2019;**120**:883–95. doi:10.1038/s41416-019-0438-1

4 MacMillan RD, James R, Gale KL, *et al.* Therapeutic mammaplasty. *J Surg Oncol* Published Online First: 2014. doi:10.1002/jso.23659

5 McIntosh J, O'Donoghue JM. Therapeutic mammaplasty - A systematic review of the evidence. *Eur J Surg Oncol* 2012;**38**:196–202. doi:10.1016/j.ejso.2011.12.004

6 Mansfield L, Agrawal A, Cutress RI. Oncoplastic breast conserving surgery. *Gland Surg* Published Online First: 2013. doi:10.3978/j.issn.2227-684X.2013.08.04

7 Piper ML, Sbitany H. Contemporary Strategies in Breast Reconstruction. *Am J Hematol Oncol* 2015;**11**:31–7.http://search.ebscohost.com/login.aspx?direct=true&db=cin20&AN=143179692&site=ehost-live

8 Bamford R, Sutton R, McIntosh J. Therapeutic mammoplasty allows for clear surgical margins in large and multifocal tumours without delaying adjuvant therapy. *Breast* Published Online First: 2015. doi:10.1016/j.breast.2015.01.003

9 Williamson PR, Altman DG, Bagley H, *et al.* The COMET Handbook: Version 1.0. *Trials* 2017;**18**:1–50. doi:10.1186/s13063-017-1978-4

10 Potter S, Holcombe C, Ward JA, *et al.* Development of a core outcome set for research and

For peer review only

audit studies in reconstructive breast surgery. *Br J Surg* 2015;**102**:1360–71. doi:10.1002/bjs.9883

11 Moher D, Shamseer L, Clarke M, *et al.* Preferred reporting items for systematic review and meta-analysis protocols (PRISMA-P) 2015 statement. *Syst Rev* 2015;**4**:1. doi:10.1186/2046-4053-4-1

12 Baker E, Kim B, Rattay T, *et al.* The TeaM (Therapeutic Mammaplasty) study: Protocol for a prospective multi-centre cohort study to evaluate the practice and outcomes of therapeutic mammaplasty. *Int J Surg Protoc* 2016;**1**:3–10. doi:10.1016/j.isjp.2016.08.001

13 National Mastectomy and Breast Reconstruction Audit. Fourth Annual Report. 2011.

14 Rainsbury D, Willett A. Oncoplastic Breast Reconstruction: Guidelines for Best Practice. Association of breast Surgery and British Association of Plastic & Reconstructive and Aesthetic Surgeons. 2012.

15 *Cochrane Handbook for Systematic Reviews of Interventions*. 2019. doi:10.1002/9781119536604

16 The InterTASC Information Specialists' Sub-Group (ISSG) Search Filters Resource: https://sites.google.com/a/york.ac.uk/issg-search-filters-resource/ (accessed 11 Feb 2019).

17 Liberati A, Altman DG, Tetzlaff J, *et al.* The PRISMA statement for reporting systematic reviews and meta-analyses of studies that evaluate health care interventions: explanation and elaboration. *J Clin Epidemiol* 2009;**62**:e1–34. doi:10.1016/j.jclinepi.2009.06.006

For peer review only

For peer review only - http://bmjopen.bmj.com/site/about/guidelines.xhtml

Data statement

Data from the systematic review described in this protocol will be published in a peer-reviewed journal. Raw data can be requested from the corresponding author in due course.

Conflicts of interest: The authors have no conflicts of interest to declare.

Acknowledgements

The authors gratefully acknowledge Mr Michael Gainsford (St Mary's Hospital Library, Imperial College London) for his support developing the literature searches for this systematic review.

Authors' contributions

AL generated the systematic review question, design and search terms. AL drafted the protocol manuscript. DL and RK critically reviewed the systematic review question and design and reviewed this protocol manuscript.

Funding statement

This work is independent research funded by the National Institute for Health Research (NIHR) Imperial Biomedical Research Centre (BRC). The views expressed in this publication are those of the authors and not necessarily those of the NHS, the NIHR or the Department of Health.

For peer review only

Tables

Table 1. Inclusion and exclusion criteria for the systematic review.

Inclusion criteria	Exclusion criteria
• Randomised and non-randomised trials, cohort studies and case-control studies<br>• Adult female participants undergoing TM as primary treatment for breast cancer (including both immediate and delayed symmetrisation)<br>• TM techniques may include, but are not limited to, Wise pattern reduction; vertical scar, Benelli and tennis racket mammoplasty; Grisotti flaps and horizontal wedge excision	• Wrong study design: systematic reviews, meta-analyses, case series, case reports, conference abstracts, and animal, cadaveric or laboratory studies<br>• Non-English language articles<br>• Non-oncological breast surgery<br>• Studies which do not report TM techniques (including total mastectomy with or without reconstruction/ symmetrisation or standard BCS)<br>• Studies with male participants or those who are < 16 years old<br>• Articles which do not report patient outcomes<br>• BCS combined with volume replacement procedures including but not limited to: use of implants, latissimus dorsi mini-flaps, thoracodorsal artery perforator flaps and lateral intercostal artery perforator flaps

Abbreviation: BCS, breast conserving surgery; TM, therapeutic mammaplasty

For peer review only - http://bmjopen.bmj.com/site/about/guidelines.xhtml

Table 2. Example search strategy for Ovid Medline.

Search concept		
Therapeutic mammaplasty	Breast cancer	Study design
1. (therapeutic adj3 mamm?plast*).mp.	6. exp Breast Neoplasms/	12. randomized controlled trial.pt.
2. reduction mamm?plast*.mp.	7. breast neoplasm*.mp.	13. controlled clinical trial.pt.
3. oncoplastic breast surger*.mp.	8. (breast adj2 cancer*).mp.	14. randomi?ed.ab.
4. Mammaplasty/	9. (breast adj2 tumo?r*).mp.	15. placebo.ab.
5. 1 or 2 or 3 or 4	10. 6 or 7 or 8 or 9	16. drug therapy.fs.
		17. randomly.ab.
		18. trial.ab.
		19. groups.ab.
		20. 12 or 13 or 14 or 15 or 16 or 17 or 18 or 19
		21. exp cohort studies/
		22. cohort$.tw.
		23. controlled clinical trial.pt.
		24. epidemiologic methods/
		25. limit 24 to yr="1966 - 1989"
		26. exp case-control studies/
		27. (case$ and control$).tw.
		28. 21 or 22 or 23
		29. 24 or 25 or 26 or 27
		30. 28 or 29

For peer review only

For peer review only - http://bmjopen.bmj.com/site/about/guidelines.xhtml

11. Combined search for therapeutic mammaplasty AND breast cancer = (5 AND 10)	31. Trial OR cohort study OR case-control study= (20 OR 30)
Overall search = (11 AND 31)	

Word count: 1442 (max 4000)

For peer review only

PRISMA-P (Preferred Reporting Items for Systematic review and Meta-Analysis Protocols) 2015 checklist: recommended items to address in a systematic review protocol*

Section and topic	Item No	Checklist item	Page reference in this protocol
**ADMINISTRATIVE INFORMATION**			
Title:			1
Identification	1a	Identify the report as a protocol of a systematic review	
Update	1b	If the protocol is for an update of a previous systematic review, identify as such	
Registration	2	If registered, provide the name of the registry (such as PROSPERO) and registration number	3
Authors:			
Contact	3a	Provide name, institutional affiliation, e-mail address of all protocol authors; provide physical mailing address of corresponding author	1
Contributions	3b	Describe contributions of protocol authors and identify the guarantor of the review	17
Amendments	4	If the protocol represents an amendment of a previously completed or published protocol, identify as such and list changes; otherwise, state plan for documenting important protocol amendments	n/a
Support:			
Sources	5a	Indicate sources of financial or other support for the review	n/a- no funding
Sponsor	5b	Provide name for the review funder and/or sponsor	
Role of sponsor or funder	5c	Describe roles of funder(s), sponsor(s), and/or institution(s), if any, in developing the protocol	
**INTRODUCTION**			
Rationale	6	Describe the rationale for the review in the context of what is already known	5-6
Objectives	7	Provide an explicit statement of the question(s) the review will address with reference to participants, interventions, comparators, and outcomes (PICO)	6
**METHODS**			
Eligibility criteria	8	Specify the study characteristics (such as PICO, study design, setting, time frame) and report characteristics (such as years considered, language, publication status) to be used as criteria for eligibility for the review	8
Information sources	9	Describe all intended information sources (such as electronic databases, contact with study authors, trial registers or other grey literature sources) with planned dates of coverage	8
Search strategy	10	Present draft of search strategy to be used for at least one electronic database, including planned limits, such that it could be repeated	9

For peer review only

Study records:			9
Data management	11a	Describe the mechanism(s) that will be used to manage records and data throughout the review	
Selection process	11b	State the process that will be used for selecting studies (such as two independent reviewers) through each phase of the review (that is, screening, eligibility and inclusion in meta-analysis)	
Data collection process	11c	Describe planned method of extracting data from reports (such as piloting forms, done independently, in duplicate), any processes for obtaining and confirming data from investigators	
Data items	12	List and define all variables for which data will be sought (such as PICO items, funding sources), any pre-planned data assumptions and simplifications	
Outcomes and prioritization	13	List and define all outcomes for which data will be sought, including prioritization of main and additional outcomes, with rationale	10
Risk of bias in individual studies	14	Describe anticipated methods for assessing risk of bias of individual studies, including whether this will be done at the outcome or study level, or both; state how this information will be used in data synthesis	n/a
Data synthesis	15a	Describe criteria under which study data will be quantitatively synthesised	10
	15b	If data are appropriate for quantitative synthesis, describe planned summary measures, methods of handling data and methods of combining data from studies, including any planned exploration of consistency (such as I^2, Kendall's τ)	
	15c	Describe any proposed additional analyses (such as sensitivity or subgroup analyses, meta-regression)	
	15d	If quantitative synthesis is not appropriate, describe the type of summary planned	
Meta-bias(es)	16	Specify any planned assessment of meta-bias(es) (such as publication bias across studies, selective reporting within studies)	n/a
Confidence in cumulative evidence	17	Describe how the strength of the body of evidence will be assessed (such as GRADE)	n/a

*** It is strongly recommended that this checklist be read in conjunction with the PRISMA-P Explanation and Elaboration (cite when available) for important clarification on the items. Amendments to a review protocol should be tracked and dated. The copyright for PRISMA-P (including checklist) is held by the PRISMA-P Group and is distributed under a Creative Commons Attribution Licence 4.0.**

From: Shamseer L, Moher D, Clarke M, Ghersi D, Liberati A, Petticrew M, Shekelle P, Stewart L, PRISMA-P Group. Preferred reporting items for systematic review and meta-analysis protocols (PRISMA-P) 2015: elaboration and explanation. BMJ. 2015 Jan 2;349(jan02 1):g7647.

For peer review only

Supplementary material: Search strategies

Ovid EMBASE

1. exp breast cancer/
2. breast neoplasm*.mp.
3. (breast adj2 cancer*).mp.
4. (breast adj2 tumo?r*).mp.
5. 1 or 2 or 3 or 4
6. (therapeutic adj3 mamm?plast*).mp.
7. reduction mamm?plast*.mp.
8. oncoplastic breast surger*.mp.
9. breast reconstruction/
10. 6 or 7 or 8 or 9
11. 5 and 10
12. clinical trial.de.
13. randomization.de.
14. crossover procedure.de.
15. randomized controlled trial.de.
16. single blind procedure.de.
17. double blind procedure.de.
18. placebo.de.
19. prospective study.de.
20. (randomi?ed controlled adj1 trial*).mp. [mp=title, abstract, heading word, drug trade name, original title, device manufacturer, drug manufacturer, device trade name, keyword, floating subheading word, candidate term word]
21. rct.mp. [mp=title, abstract, heading word, drug trade name, original title, device manufacturer, drug manufacturer, device trade name, keyword, floating subheading word, candidate term word]
22. (random* adj1 allocat*).mp. [mp=title, abstract, heading word, drug trade name, original title, device manufacturer, drug manufacturer, device trade name, keyword, floating subheading word, candidate term word]
23. (single adj1 blind*).mp. [mp=title, abstract, heading word, drug trade name, original title, device manufacturer, drug manufacturer, device trade name, keyword, floating subheading word, candidate term word]

For peer review only

24. (double adj1 blind*).mp. [mp=title, abstract, heading word, drug trade name, original title, device manufacturer, drug manufacturer, device trade name, keyword, floating subheading word, candidate term word]
25. ((treble or triple) adj1 (blind* or placebo*)).mp. [mp=title, abstract, heading word, drug trade name, original title, device manufacturer, drug manufacturer, device trade name, keyword, floating subheading word, candidate term word]
26. 12 or 13 or 14 or 15 or 16 or 17 or 18 or 19 or 20 or 21 or 22 or 23 or 24 or 25
27. exp cohort analysis/
28. exp longitudinal study/
29. exp prospective study/
30. exp follow up/
31. cohort$.tw.
32. exp case control study/
33. (case$ and control$).tw.
34. 27 or 28 or 29 or 30 or 31 or 32 or 33
35. 26 or 34
36. 11 and 35

Ovid Medline

1. (therapeutic adj3 mamm?plast*).mp.
2. reduction mamm?plast*.mp.
3. oncoplastic breast surger*.mp.
4. Mammaplasty/
5. 1 or 2 or 3 or 4
6. exp Breast Neoplasms/
7. breast neoplasm*.mp.
8. (breast adj2 cancer*).mp.
9. (breast adj2 tumo?r*).mp.
10. 6 or 7 or 8 or 9
11. 5 and 10
12. randomized controlled trial.pt.
13. controlled clinical trial.pt.
14. randomi?ed.ab.

For peer review only

15. placebo.ab.

16. drug therapy.fs.

17. randomly.ab.

18. trial.ab.

19. groups.ab.

20. 12 or 13 or 14 or 15 or 16 or 17 or 18 or 19

21. exp cohort studies/

22. cohort$.tw.

23. controlled clinical trial.pt.

24. epidemiologic methods/

25. limit 24 to yr="1966 - 1989"

26. exp case-control studies/

27. (case$ and control$).tw.

28. 21 or 22 or 23

29. 24 or 25 or 26 or 27

30. 28 or 29

31. 20 or 30

32. 11 and 31

CINAHL

1. MH Breast Neoplasms+
2. Breast neoplasm*.mp
3. (breast adj2 cancer*).mp.
4. 1 or 2 or 3
5. therapeutic adj3 mamm?plast*.mp
6. oncoplastic breast surger*
7. reduction mamm?plast*
8. breast reconstruction/
9. 5 or 6 or 7 or 8
10. 4 and 9

Web of Science

For peer review only

ALL=((breast neoplasm* OR (breast "NEAR" cancer*) OR (breast "NEAR" tumo?r*)) AND (therapeutic "NEAR" mamm?plast* OR reduction mamm?plast* OR oncoplastic breast surger*))

For peer review only

BMJ Open

Outcomes and outcome measures reported in clinical studies of therapeutic mammaplasty: A systematic review protocol

Journal:	*BMJ Open*
Manuscript ID	bmjopen-2020-046438.R1
Article Type:	Protocol
Date Submitted by the Author:	06-Apr-2021

Complete List of Authors:	Lee, Alice; Imperial College London, Department of Surgery and Cancer<br>Kwasnicki, Richard; Imperial College London, Department of Surgery and Cancer<br>Leff, Daniel; Imperial College London, Department of Surgery and Cancer

**Primary Subject Heading**:	Surgery
Secondary Subject Heading:	Evidence based practice
Keywords:	Plastic & reconstructive surgery < SURGERY, Breast surgery < SURGERY, STATISTICS & RESEARCH METHODS

SCHOLARONE™
Manuscripts

I, the Submitting Author has the right to grant and does grant on behalf of all authors of the Work (as defined in the below author licence), an exclusive licence and/or a non-exclusive licence for contributions from authors who are: i) UK Crown employees; ii) where BMJ has agreed a CC-BY licence shall apply, and/or iii) in accordance with the terms applicable for US Federal Government officers or employees acting as part of their official duties; on a worldwide, perpetual, irrevocable, royalty-free basis to BMJ Publishing Group Ltd ("BMJ") its licensees and where the relevant Journal is co-owned by BMJ to the co-owners of the Journal, to publish the Work in this journal and any other BMJ products and to exploit all rights, as set out in our licence.

The Submitting Author accepts and understands that any supply made under these terms is made by BMJ to the Submitting Author unless you are acting as an employee on behalf of your employer or a postgraduate student of an affiliated institution which is paying any applicable article publishing charge ("APC") for Open Access articles. Where the Submitting Author wishes to make the Work available on an Open Access basis (and intends to pay the relevant APC), the terms of reuse of such Open Access shall be governed by a Creative Commons licence – details of these licences and which Creative Commons licence will apply to this Work are set out in our licence referred to above.

Other than as permitted in any relevant BMJ Author's Self Archiving Policies, I confirm this Work has not been accepted for publication elsewhere, is not being considered for publication elsewhere and does not duplicate material already published. I confirm all authors consent to publication of this Work and authorise the granting of this licence.

For peer review only

Outcomes and outcome measures reported in clinical studies of therapeutic mammaplasty: A systematic review protocol

Alice Lee[1], Richard M Kwasnicki[1], Daniel Leff[1]

Author affiliations:

1. Department of Surgery and Cancer, Faculty of Medicine, Imperial College London, South Kensington Campus, London SW7 2AZ, UK

Corresponding author:

Dr Alice Lee

Address: Department of Surgery and Cancer, Faculty of Medicine, Imperial College London, South Kensington Campus, London SW7 2AZ, UK

Email: alice.lee13@imperial.ac.uk

For peer review only

Abstract

Introduction

Therapeutic mammaplasty (TM) is an oncological procedure which combines tumour resection with breast reduction and mastopexy techniques. Previous systematic reviews have demonstrated oncological safety of TM, but poor and inconsistent reporting of quality of life, aesthetic and functional outcomes, often with non-validated measurement tools. Moreover, there is a paucity of patient-reported outcome measures. Standardisation of outcome reporting is required to enable study results to be compared and combined, for example through Core Outcome Set (COS) development. This systematic review aims to comprehensively describe the outcomes reported in clinical studies of TM, their respective outcome measures and the time points at which they were evaluated. The overall objective is to facilitate the development of a COS for TM.

Methods and analysis

A systematic review of clinical studies evaluating outcomes following TM will be completed according to the Preferred Reporting Items for Systematic Reviews and Meta-Analyses (PRISMA) guidelines. The following electronic databases have been searched from inception to 05/08/2020: Ovid Medline, EMBASE, CINAHL and Web of Science. Primary outcomes will include the number of reported outcomes of various types (clinical, aesthetic, functional, quality-of-life and cost-effectiveness), whether these are patient- or clinician-reported, how outcomes are defined and the outcome measurement tool(s) used. The time point(s) at which outcomes were measured will be a secondary outcome. No studies will be excluded on the basis of methodological quality in order to generate a comprehensive list of reported outcomes and outcome measures; hence, risk of bias assessment is not required. The data will be described narratively. This protocol has been reported in line with PRISMA-P.

For peer review only

For peer review only - http://bmjopen.bmj.com/site/about/guidelines.xhtml

Ethics and dissemination

This study does not involve human or animal participants, hence ethical approval is not required. The findings will be published in a peer-reviewed journal and presented at relevant conferences.

Registration: This review is registered in PROSPERO (CRD42020200365).

Word count: 300

Keywords: therapeutic mammaplasty; core outcome set; outcome measurement; patient-reported outcomes

For peer review only

Article Summary

Strengths and limitations of this study (up to 5 bullet points)

- This systematic review will facilitate the development of the first Core Outcome Set for therapeutic mammaplasty.
- A Core Outcome Set will improve the synthesis and meta-analysis of new and existing research into therapeutic mammaplasty.
- The systematic review described in this protocol will build on existing literature by providing an updated review of outcomes following therapeutic mammaplasty with a broader scope, including clinical, aesthetic, functional, quality-of-life and patient-reported outcomes.
- The literature search was limited to English language articles, which may have excluded some relevant papers.

For peer review only

For peer review only - http://bmjopen.bmj.com/site/about/guidelines.xhtml

Introduction

Background

Therapeutic mammaplasty (TM) is an oncological procedure which combines cancer resection with breast reduction and mastopexy techniques [1]. TM can facilitate breast-conserving surgery (BCS) for larger tumours [2] to safely avoid mastectomy [3] and improve cosmesis in cases where standard BCS would otherwise have poor outcomes [4]. TM may also minimise radiotherapy-related side effects in women with larger breasts [4,5] and be a favourable option for women with pre-existing macromastia who seek the functional and psychological benefits of breast reduction surgery [4]. Previous systematic reviews have suggested satisfactory oncological safety of TM [2,4,6], but poor and inconsistent reporting of quality of life, aesthetic and functional outcomes, often with non-validated measurement tools [5,7,8]. Moreover, there is a paucity of patient-reported outcome measures [6].

A Core Outcome Set (COS) describes the minimum number of outcomes to be reported across all trials of one healthcare domain [9]. This reduces the heterogeneity of outcome reporting across trials, allowing results to be compared and combined in meta-analyses, to inform best medical practice [9]. TM is becoming routine practice in oncoplastic breast units, however there is no standardised way to evaluate outcomes following this procedure which incorporates the views of both healthcare professionals and patients as stakeholders. A related COS on reconstructive breast surgery [10] mainly focussed on post-mastectomy reconstruction; only 10% of patient stakeholders in the project had undergone TM and some outcomes included in the final COS (e.g., implant related complications) are less relevant to the TM population. There is good reason to hypothesise that TM patients may evaluate and prioritise their treatment outcomes differently to patients undergoing other forms of breast reconstruction. For example, improved functional outcomes associated with breast reduction techniques and avoidance of mastectomy may significantly drive treatment decisions.

For peer review only

Rationale

In order to develop a COS for TM, a comprehensive systematic review of all available outcomes and outcome measures reported in the literature is required. The systematic review described in this protocol is the first stage in the development of a COS for TM, which is planned and has been prospectively registered on the Core Outcome Measures in Effectiveness Trials (COMET) database (http://comet-initiative.org/Studies/Details/1655).

Aims and objectives

Review aims

The overall aim of this systematic review is to identify all outcomes and outcome measures used to evaluate TM in the literature and how the authors define these outcomes. The timepoints at which these outcomes are measured will be a secondary outcome.

Objectives

The specific objectives of this review are to analyse all clinical studies of TM in adult, female participants in order to:

i) Identify the number of unique outcomes and outcome measures reported;
ii) Identify and describe variation in outcome definitions;
iii) Identify and describe variation in the timepoint(s) used to measure outcomes;
iv) Identify the number of different types of outcome (clinical, aesthetic, functional, quality-of-life and cost-effectiveness outcomes) reported per study and across all included studies and whether these are clinician- or patient-reported;
v) Group unique outcomes into domains, to facilitate the development of a COS for TM.

For peer review only

Methods and analysis

This protocol has been developed in accordance with the PRISMA-P guidelines [11]. A PRISMA-P checklist for this protocol can be found in **supplementary material 1**. The systematic review described in this protocol has been prospectively registered on PROSPERO (CRD42020200365). Any amendments to this protocol, involving screening, data extraction or data synthesis will be documented and referenced in any subsequent, related publications.

Eligibility criteria

This systematic review will include clinical studies of adult, female participants who have undergone TM as primary treatment for breast cancer. For the purpose of this systematic review, TM will be defined as the use of oncoplastic reduction or mastopexy techniques, including removal of the skin envelope and/or nipple if indicated, to treat pre-invasive or invasive breast cancer with BCS [12]. The eligibility criteria are summarised in **Table 1**. At the time of writing this protocol, screening (by title and abstract) has commenced. The initial search returned 5709 de-duplicated articles.

Information sources

The following electronic databases have been searched from inception to 05/08/2020: Ovid Medline, EMBASE, CINAHL and Web of Science. The reference lists of included articles will also be hand-searched. The outcomes generated from this review will be cross-referenced with those reported in the Oncoplastic Breast Reconstruction Guidelines for Best Practice co-produced by the Association of Breast Surgery and British Association of Plastic Reconstructive and Aesthetic Surgeons [13]. Outcomes from these national documents which are relevant to the TM population and not already included in the review will be added.

Search strategy

For peer review only

For peer review only - http://bmjopen.bmj.com/site/about/guidelines.xhtml

An example search strategy for Ovid Medline is provided in **Table 2**. The search strategies for EMBASE, CINAHL and Web of Science can be found in **supplementary material 2**. In order to focus the search and make screening numbers manageable, validated study design filters for clinical trials, cohort studies and case-control studies were used [14,15].

Study records

Covidence software (v2103) will be used for de-duplication of citations and article screening. Two researchers will independently screen papers against inclusion criteria in two stages: i) by title and abstract, and ii) by full text. Data will be extracted using a piloted form in Microsoft Excel (v16.41), developed for the purposes of this review. Included studies will be extracted independently by two researchers. The first 10 included full texts will be extracted to trial the suitability of the data extraction sheet. Any conflicts which arise in the screening or extraction stages will be resolved through discussion between the relevant researchers; if they cannot be resolved a third researcher (RMK or DL) will make the final decision.

Data items

The following variables will be extracted: study details (publication year, study design, TM procedure, average follow-up time) and information on study population (n number and average age). Outcome data will include total number of reported outcomes, whether outcomes are clinician- or patient-reported, how study authors have defined each outcome, the outcome measure(s) used and the time point(s) at which each outcome is measured. Outcomes will be categorised (into clinical, aesthetic, functional, quality-of-life and cost-effectiveness outcomes) by the researchers performing data extraction. Clinical outcomes will include oncological outcomes and operative complications. Aesthetic outcomes will include all measures of satisfaction with post-operative appearance, either clinician- or patient-reported. Functional outcomes will include, but are not limited to, level of physical activity, neck, shoulder, breast or back pain and intertrigo. Quality-of-life outcomes will include

For peer review only

domains such as psychosocial, sexual and physical wellbeing. Cost-effectiveness outcomes will include formal analysis of cost-effectiveness or surrogate measures such as length of stay. Outcome measures which fall into more than one of the aforementioned categories will be reported as such and described narratively.

Outcomes and prioritisation

The primary outcomes of this study will include the number of unique outcomes and outcome measures reported in studies of TM and any reported variation in outcome definition between studies. As a secondary outcome, we will summarise the timepoints used for outcome measurement and any variation between studies with respect to the timing of outcome measurement.

Risk of bias in individual studies

No studies will be excluded on the basis of methodological quality in order to generate a comprehensive list of reported outcomes and outcome measures; hence, risk of bias assessment is not required.

Data synthesis

The following data will be summarised quantitively: number of outcomes categorised as clinical, aesthetic, quality-of-life and cost-effectiveness per study; the number of different definitions used for each outcome across all included studies and the number of different outcome measures and time points used to measure each outcome across all included studies. The percentage of included studies which evaluate aesthetic, functional, quality-of-life, cost-effectiveness and patient-reported outcomes will be calculated. Outcomes will then be grouped into domains using an existing [9] or author-generated ontological framework, depending on the final list of outcomes obtained.

For peer review only

For peer review only - http://bmjopen.bmj.com/site/about/guidelines.xhtml

Patient and public involvement

Patients were not directly involved in the design or conduct of this systematic review, since no participant recruitment will take place and the research is based on previously published data.

Ethics and dissemination

This study does not involve human or animal participants, hence ethical approval is not required. The completed systematic review will be presented at relevant academic conferences, reported in accordance with PRISMA guidelines [16] and published in a peer-reviewed journal.

Data statement

Data from the systematic review described in this protocol will be published in a peer-reviewed journal. Raw data can be requested from the corresponding author in due course.

Acknowledgements

The authors gratefully acknowledge Mr Michael Gainsford (St Mary's Hospital Library, Imperial College London) for his support developing the literature searches for this systematic review.

Contributorship statement

AL generated the systematic review question, design and search terms. AL drafted the protocol manuscript. DL and RK critically reviewed the systematic review question and design and reviewed this protocol manuscript.

Conflicts of interest: The authors have no conflicts of interest to declare.

Funding statement

For peer review only

For peer review only - http://bmjopen.bmj.com/site/about/guidelines.xhtml

This work is independent research funded by the National Institute for Health Research (NIHR) Imperial Biomedical Research Centre (BRC). The views expressed in this publication are those of the authors and not necessarily those of the NHS, the NIHR or the Department of Health.

For peer review only

References

1 Currie A, Chong K, Davies GL. Using therapeutic mammoplasty to extend the role of breast-conserving surgery in women with larger or ptotic breasts. *Ann R Coll Surg Engl* Published Online First: 2013. doi:10.1308/003588413X13511609958091

2 Schaverien M V., Raine C, Majdak-Paredes E, *et al.* Therapeutic mammaplasty-Extending indications and achieving low incomplete excision rates. *Eur J Surg Oncol* Published Online First: 2013. doi:10.1016/j.ejso.2013.01.006

3 O'Connell RL, Rattay T, Dave R V, *et al.* The impact of immediate breast reconstruction on the time to delivery of adjuvant therapy: the iBRA-2 study. *Br J Cancer* 2019;**120**:883–95. doi:10.1038/s41416-019-0438-1

4 MacMillan RD, James R, Gale KL, *et al.* Therapeutic mammaplasty. *J Surg Oncol* Published Online First: 2014. doi:10.1002/jso.23659

5 McIntosh J, O'Donoghue JM. Therapeutic mammaplasty - A systematic review of the evidence. *Eur J Surg Oncol* 2012;**38**:196–202. doi:10.1016/j.ejso.2011.12.004

6 Mansfield L, Agrawal A, Cutress RI. Oncoplastic breast conserving surgery. *Gland Surg* Published Online First: 2013. doi:10.3978/j.issn.2227-684X.2013.08.04

7 Piper ML, Sbitany H. Contemporary Strategies in Breast Reconstruction. *Am J Hematol Oncol* 2015;**11**:31–7.http://search.ebscohost.com/login.aspx?direct=true&db=cin20&AN=143179692&site=ehost-live

8 Bamford R, Sutton R, McIntosh J. Therapeutic mammoplasty allows for clear surgical margins in large and multifocal tumours without delaying adjuvant therapy. *Breast* Published Online First: 2015. doi:10.1016/j.breast.2015.01.003

9 Williamson PR, Altman DG, Bagley H, *et al.* The COMET Handbook: Version 1.0. *Trials* 2017;**18**:1–50. doi:10.1186/s13063-017-1978-4

10 Potter S, Holcombe C, Ward JA, *et al.* Development of a core outcome set for research and

For peer review only

For peer review only - http://bmjopen.bmj.com/site/about/guidelines.xhtml

audit studies in reconstructive breast surgery. *Br J Surg* 2015;**102**:1360–71. doi:10.1002/bjs.9883

11 Moher D, Shamseer L, Clarke M, *et al.* Preferred reporting items for systematic review and meta-analysis protocols (PRISMA-P) 2015 statement. *Syst Rev* 2015;**4**:1. doi:10.1186/2046-4053-4-1

12 Baker E, Kim B, Rattay T, *et al.* The TeaM (Therapeutic Mammaplasty) study: Protocol for a prospective multi-centre cohort study to evaluate the practice and outcomes of therapeutic mammaplasty. *Int J Surg Protoc* 2016;**1**:3–10. doi:10.1016/j.isjp.2016.08.001

13 Rainsbury D, Willett A. Oncoplastic Breast Reconstruction: Guidelines for Best Practice. Association of breast Surgery and British Association of Plastic & Reconstructive and Aesthetic Surgeons. 2012.

14 *Cochrane Handbook for Systematic Reviews of Interventions*. 2019. doi:10.1002/9781119536604

15 The InterTASC Information Specialists' Sub-Group (ISSG) Search Filters Resource: https://sites.google.com/a/york.ac.uk/issg-search-filters-resource/ (accessed 11 Feb 2019).

16 Liberati A, Altman DG, Tetzlaff J, *et al.* The PRISMA statement for reporting systematic reviews and meta-analyses of studies that evaluate health care interventions: explanation and elaboration. *J Clin Epidemiol* 2009;**62**:e1–34. doi:10.1016/j.jclinepi.2009.06.006

For peer review only

Tables

Table 1. Inclusion and exclusion criteria for the systematic review.

Inclusion criteria	Exclusion criteria
• Randomised and non-randomised trials, cohort studies and case-control studies<br>• Adult female participants undergoing TM as primary treatment for breast cancer (including both immediate and delayed symmetrisation)<br>• TM techniques (level 1-2 oncoplastic breast surgery) including the following skin incision patterns: Wise, vertical scar, peri- or circumareolar, Grisotti, melon slice (horizontal wedge excision)	• Wrong study design: systematic reviews, meta-analyses, case series, case reports, conference abstracts, and animal, cadaveric or laboratory studies<br>• Non-English language articles<br>• Non-oncological breast surgery<br>• Studies which do not report TM techniques (including total mastectomy with or without reconstruction/ symmetrisation or standard BCS)<br>• Studies with male participants or those who are < 18 years old<br>• Articles which do not report patient outcomes<br>• BCS combined with volume replacement procedures including but not limited to implants, latissimus dorsi mini-flaps, thoracodorsal artery perforator flaps, lateral intercostal artery perforator flaps

Abbreviation: BCS, breast conserving surgery; TM, therapeutic mammaplasty

Table 2. Example search strategy for Ovid Medline.

For peer review only

Search concept		
Therapeutic mammaplasty	Breast cancer	Study design
1. (therapeutic adj3 mamm?plast*).mp.<br>2. reduction mamm?plast*.mp.<br>3. oncoplastic breast surger*.mp.<br>4. Mammaplasty/<br>5. 1 or 2 or 3 or 4	6. exp Breast Neoplasms/<br>7. breast neoplasm*.mp.<br>8. (breast adj2 cancer*).mp.<br>9. (breast adj2 tumo?r*).mp.<br>10. 6 or 7 or 8 or 9	12. randomized controlled trial.pt.<br>13. controlled clinical trial.pt.<br>14. randomi?ed.ab.<br>15. placebo.ab.<br>16. drug therapy.fs.<br>17. randomly.ab.<br>18. trial.ab.<br>19. groups.ab.<br>20. 12 or 13 or 14 or 15 or 16 or 17 or 18 or 19<br>21. exp cohort studies/<br>22. cohort$.tw.<br>23. controlled clinical trial.pt.<br>24. epidemiologic methods/<br>25. limit 24 to yr="1966 - 1989"<br>26. exp case-control studies/<br>27. (case$ and control$).tw.<br>28. 21 or 22 or 23<br>29. 24 or 25 or 26 or 27<br>30. 28 or 29

For peer review only - http://bmjopen.bmj.com/site/about/guidelines.xhtml

11. Combined search for therapeutic mammaplasty AND breast cancer = (5 AND 10)	31. Trial OR cohort study OR case-control study= (20 OR 30)
Overall search = (11 AND 31)	

Word count: 1511 (max 4000)

For peer review only

PRISMA-P (Preferred Reporting Items for Systematic review and Meta-Analysis Protocols) 2015 checklist: recommended items to address in a systematic review protocol*

Section and topic	Item No	Checklist item	Page reference in this protocol
**ADMINISTRATIVE INFORMATION**			
Title:			1
Identification	1a	Identify the report as a protocol of a systematic review	
Update	1b	If the protocol is for an update of a previous systematic review, identify as such	
Registration	2	If registered, provide the name of the registry (such as PROSPERO) and registration number	3
Authors:			
Contact	3a	Provide name, institutional affiliation, e-mail address of all protocol authors; provide physical mailing address of corresponding author	1
Contributions	3b	Describe contributions of protocol authors and identify the guarantor of the review	17
Amendments	4	If the protocol represents an amendment of a previously completed or published protocol, identify as such and list changes; otherwise, state plan for documenting important protocol amendments	n/a
Support:			
Sources	5a	Indicate sources of financial or other support for the review	n/a- no funding
Sponsor	5b	Provide name for the review funder and/or sponsor	
Role of sponsor or funder	5c	Describe roles of funder(s), sponsor(s), and/or institution(s), if any, in developing the protocol	
**INTRODUCTION**			
Rationale	6	Describe the rationale for the review in the context of what is already known	5-6
Objectives	7	Provide an explicit statement of the question(s) the review will address with reference to participants, interventions, comparators, and outcomes (PICO)	6
**METHODS**			
Eligibility criteria	8	Specify the study characteristics (such as PICO, study design, setting, time frame) and report characteristics (such as years considered, language, publication status) to be used as criteria for eligibility for the review	8
Information sources	9	Describe all intended information sources (such as electronic databases, contact with study authors, trial registers or other grey literature sources) with planned dates of coverage	8
Search strategy	10	Present draft of search strategy to be used for at least one electronic database, including planned limits, such that it could be repeated	9

For peer review only

Study records:			9
Data management	11a	Describe the mechanism(s) that will be used to manage records and data throughout the review	
Selection process	11b	State the process that will be used for selecting studies (such as two independent reviewers) through each phase of the review (that is, screening, eligibility and inclusion in meta-analysis)	
Data collection process	11c	Describe planned method of extracting data from reports (such as piloting forms, done independently, in duplicate), any processes for obtaining and confirming data from investigators	
Data items	12	List and define all variables for which data will be sought (such as PICO items, funding sources), any pre-planned data assumptions and simplifications	
Outcomes and prioritization	13	List and define all outcomes for which data will be sought, including prioritization of main and additional outcomes, with rationale	10
Risk of bias in individual studies	14	Describe anticipated methods for assessing risk of bias of individual studies, including whether this will be done at the outcome or study level, or both; state how this information will be used in data synthesis	n/a
Data synthesis	15a	Describe criteria under which study data will be quantitatively synthesised	10
	15b	If data are appropriate for quantitative synthesis, describe planned summary measures, methods of handling data and methods of combining data from studies, including any planned exploration of consistency (such as I^2, Kendall's τ)	
	15c	Describe any proposed additional analyses (such as sensitivity or subgroup analyses, meta-regression)	
	15d	If quantitative synthesis is not appropriate, describe the type of summary planned	
Meta-bias(es)	16	Specify any planned assessment of meta-bias(es) (such as publication bias across studies, selective reporting within studies)	n/a
Confidence in cumulative evidence	17	Describe how the strength of the body of evidence will be assessed (such as GRADE)	n/a

*** It is strongly recommended that this checklist be read in conjunction with the PRISMA-P Explanation and Elaboration (cite when available) for important clarification on the items. Amendments to a review protocol should be tracked and dated.** **The copyright for PRISMA-P (including checklist) is held by the PRISMA-P Group and is distributed under a Creative Commons Attribution Licence 4.0.**

From: Shamseer L, Moher D, Clarke M, Ghersi D, Liberati A, Petticrew M, Shekelle P, Stewart L, PRISMA-P Group. Preferred reporting items for systematic review and meta-analysis protocols (PRISMA-P) 2015: elaboration and explanation. BMJ. 2015 Jan 2;349(jan02 1):g7647.

For peer review only

Supplementary material: Search strategies

Ovid EMBASE

1. exp breast cancer/
2. breast neoplasm*.mp.
3. (breast adj2 cancer*).mp.
4. (breast adj2 tumo?r*).mp.
5. 1 or 2 or 3 or 4
6. (therapeutic adj3 mamm?plast*).mp.
7. reduction mamm?plast*.mp.
8. oncoplastic breast surger*.mp.
9. breast reconstruction/
10. 6 or 7 or 8 or 9
11. 5 and 10
12. clinical trial.de.
13. randomization.de.
14. crossover procedure.de.
15. randomized controlled trial.de.
16. single blind procedure.de.
17. double blind procedure.de.
18. placebo.de.
19. prospective study.de.
20. (randomi?ed controlled adj1 trial*).mp. [mp=title, abstract, heading word, drug trade name, original title, device manufacturer, drug manufacturer, device trade name, keyword, floating subheading word, candidate term word]
21. rct.mp. [mp=title, abstract, heading word, drug trade name, original title, device manufacturer, drug manufacturer, device trade name, keyword, floating subheading word, candidate term word]
22. (random* adj1 allocat*).mp. [mp=title, abstract, heading word, drug trade name, original title, device manufacturer, drug manufacturer, device trade name, keyword, floating subheading word, candidate term word]
23. (single adj1 blind*).mp. [mp=title, abstract, heading word, drug trade name, original title, device manufacturer, drug manufacturer, device trade name, keyword, floating subheading word, candidate term word]

For peer review only

24. (double adj1 blind*).mp. [mp=title, abstract, heading word, drug trade name, original title, device manufacturer, drug manufacturer, device trade name, keyword, floating subheading word, candidate term word]
25. ((treble or triple) adj1 (blind* or placebo*)).mp. [mp=title, abstract, heading word, drug trade name, original title, device manufacturer, drug manufacturer, device trade name, keyword, floating subheading word, candidate term word]
26. 12 or 13 or 14 or 15 or 16 or 17 or 18 or 19 or 20 or 21 or 22 or 23 or 24 or 25
27. exp cohort analysis/
28. exp longitudinal study/
29. exp prospective study/
30. exp follow up/
31. cohort$.tw.
32. exp case control study/
33. (case$ and control$).tw.
34. 27 or 28 or 29 or 30 or 31 or 32 or 33
35. 26 or 34
36. 11 and 35

Ovid Medline

1. (therapeutic adj3 mamm?plast*).mp.
2. reduction mamm?plast*.mp.
3. oncoplastic breast surger*.mp.
4. Mammaplasty/
5. 1 or 2 or 3 or 4
6. exp Breast Neoplasms/
7. breast neoplasm*.mp.
8. (breast adj2 cancer*).mp.
9. (breast adj2 tumo?r*).mp.
10. 6 or 7 or 8 or 9
11. 5 and 10
12. randomized controlled trial.pt.
13. controlled clinical trial.pt.
14. randomi?ed.ab.

For peer review only

15. placebo.ab.

16. drug therapy.fs.

17. randomly.ab.

18. trial.ab.

19. groups.ab.

20. 12 or 13 or 14 or 15 or 16 or 17 or 18 or 19

21. exp cohort studies/

22. cohort$.tw.

23. controlled clinical trial.pt.

24. epidemiologic methods/

25. limit 24 to yr="1966 - 1989"

26. exp case-control studies/

27. (case$ and control$).tw.

28. 21 or 22 or 23

29. 24 or 25 or 26 or 27

30. 28 or 29

31. 20 or 30

32. 11 and 31

CINAHL

1. MH Breast Neoplasms+
2. Breast neoplasm*.mp
3. (breast adj2 cancer*).mp.
4. 1 or 2 or 3
5. therapeutic adj3 mamm?plast*.mp
6. oncoplastic breast surger*
7. reduction mamm?plast*
8. breast reconstruction/
9. 5 or 6 or 7 or 8
10. 4 and 9

Web of Science

For peer review only

ALL=((breast neoplasm* OR (breast "NEAR" cancer*) OR (breast "NEAR" tumo?r*)) AND (therapeutic "NEAR" mamm?plast* OR reduction mamm?plast* OR oncoplastic breast surger*))

For peer review only
